# Impact of intraindividual pain variability on functional pain outcomes among adults with chronic pain: an ecological momentary assessment study

**DOI:** 10.1007/s10865-025-00590-x

**Published:** 2025-08-08

**Authors:** Andrew H. Rogers, Tanya Smit, Jafar Bakhshaie, Michael J. Zvolensky

**Affiliations:** 1 Division of Behavioral Medicine, Department of Medicine, Jacobs School of Medicine and Biomedical Sciences, University at Buffalo, SUNY, 462 Grider Street, Buffalo, NY 14215 USA; 2 Department of Psychology, University of Houston, Houston, TX USA; 3 Department of Psychiatry, Harvard Medical School, Massachusetts General Hospital, Boston, MA USA; 4 HEALTH Institute, University of Houston, Houston, TX USA; 5 Department of Behavioral Science, The University of Texas MD Anderson Cancer Center, Houston, TX USA

**Keywords:** Chronic pain, Pain variability, Ecological momentary assessment

## Abstract

Chronic pain is a significant public health problem linked to notable functional impairment and economic burden. Despite considerable research attention, chronic pain treatments only yield small to medium sized effects for pain complaints. Better understanding the pain experience may help improve treatment outcomes for pain. Specifically, intraindividual variation in pain intensity represents a potentially important avenue that captures the dynamic nature of pain and may improve overall understanding of pain. Limited work has examined metrics of intraindividual pain variation across chronic pain populations, but no work has examined how these metrics are associated with pain functional outcomes (pain-related disability, negative affect, and activity avoidance). Therefore, the current study utilized ecological momentary assessment to assess pain intensity, pain-related disability, negative affect, and activity avoidance, 5 times a day for 7 days, among 48 adults with chronic pain. Results suggested that pain instability metrics (e.g., intraindividual standard deviation) were associated with pain-related activity avoidance, while dynamic metrics of variability (e.g. % of time in high pain) were directly associated with pain-related disability and negative affect. Results from the current study have important clinical implications that can be applied to the assessment of pain to guide further treatment planning. Contextualizing pain as a dynamic experience that can be captured via intensive self-report assessment may improve overall intervention outcomes.

## Introduction

Chronic pain, defined as pain persisting for at least 3 months, is a major public health problem impacting over 20% of adults (Rikard et al., 2023), and leads to significant economic burden (i.e. medical expenditures, missed work days) and functional impairment (Cohen et al., 2021; Lohan et al., 2023; Turk & Patel, 2022; Yong et al., 2022). Further, chronic pain has been linked to the initiation and persistence of the opioid epidemic (Pielech et al., 2020; Witkiewitz & Vowles, 2023). Chronic pain treatments, including pharmacological and non-pharmacological treatments, yield small to medium sized effects for pain complaints, and interestingly, often show stronger effects on factors comorbid with pain, such as depression and anxiety (Hoffman et al., 2007; Malone & Strube, 1988; Niknejad et al., 2018; Shetty et al., 2024; Veehof et al., 2011). Importantly, the majority of clinical trials for chronic pain utilize single-item and/or retrospective reports of *average* pain level to assess efficacy and effectiveness, which provides a limited and potentially biased understanding of pain experience during treatment (Stone et al., 2010; Winger et al., 2019). There is a need to pursue a more nuanced understanding of pain experience among adults with chronic pain to improve clinical interventions for this population.

Within- and between-day changes in pain intensity (intraindividual pain variability) represent an important avenue to improve our understanding of pain experience (Mun et al., 2019). Theoretical work has highlighted the importance of utilizing intensive longitudinal assessment (e.g., ecological momentary assessment (EMA)) to calculate unique metrics that capture the momentary nature of pain experience above and beyond average pain level (Stone et al., 2021a, 2021b); these include the percentage of time spent in high and low pain, and how much pain tends to fluctuate for a given person, differentiating between dynamic aspects of pain and pain *instability* (Schneider et al., 2021; Stone et al., 2021a, 2021b). Thus far, several important findings have emerged in empirical work examining these pain variability metrics. For example, in one study, patients with moderate to high intra-individual pain variability (compared to low variability) reported poorer coping and maladaptive responses to pain among adults with chronic low back pain (Wesolowicz et al., 2021). In another investigation among youth with sickle cell disease, higher pain variability was associated with poorer mental health symptoms, more emergency department visits, and a greater number of healthcare visits (Pascale et al., 2023). Additionally, among adults with chronic pain, variability metrics were associated with psychosocial and functional outcomes over and above the variance accounted for by average pain level (Schneider et al., 2021). Further, some work suggests that higher baseline variability is associated with a greater pain treatment response (Harris et al., 2005; Pinto et al., 2021), whereas other work shows that variability is not related to treatment outcome (Gillving et al., 2022; Tiwari et al., 2022).

Despite the varied findings noted in past work, which may be a product of distinct methodological features of those investigations, the corpus of past work highlights that conceptualizing pain as a ‘dynamic experience’ represents an important avenue for further scientific inquiry (Mun et al., 2019). There are several gaps in the current literature. First, limited research has examined whether the range of pain variability metrics impacts *pain-related* functional outcomes (e.g., disability, activity avoidance). This information is important because it may suggest that conceptualizing pain as a dynamic experience is a clinically relevant factor for functional behavior and pain-related quality of life.

The current study utilized EMA to capture intraindividual variation in pain over 7 days among adults with chronic pain. We explored the impact of established pain variability metrics on pain-related disability, pain-related negative affect, and pain-related activity avoidance. It was hypothesized that greater pain variability would be associated with greater pain-related disability, negative affect, and activity avoidance.

## Methods

### Participants

Participants were 48 adults with self-reported chronic pain, currently enrolled in undergraduate courses at the University of Houston and elected to participate in a longitudinal observational study on pain and coping. Eligibility criteria included: (1) being between the ages of 18–65; (2) being an enrolled student at the University of Houston; (3) owning an internet-capable smartphone; and (4) currently experiencing moderate to severe idiopathic chronic pain for at least 3 months, indicated by self-report. Exclusionary criteria included: (1) endorsement of current or past psychotic spectrum symptoms, and (2) an inability to perform required self-report surveys.

### Measures

*Demographic characteristics* At baseline, participants reported on their biological sex, race, ethnicity, education, income, as well as their work current performance in relation to any time previously (not specifically anchored to pain—categorized as not working, decline in effectiveness, adequate/static, variable effectiveness, and increase in effectiveness), and social functioning (decline in competence, adequate, variable, and increase in competence).

*Pain experience* Throughout the EMA period, participants were asked to rate pain interference, pain-related negative affect, and pain-related activity avoidance. For pain interference, participants were asked how much their current pain interfered with their daily activities, on a 0 (*no interference*) to 10 (*unable to carry on any activities*) scale. For pain-related negative affect, participants were asked to report the degree they experienced anger, frustration, or depression as it relates to their current pain, rated on a scale from 0 (*not at all*) to 10 (*the most imaginable*). For pain-related activity avoidance, participants were asked, on a scale from 1 (*never true*) to 7 (*always true*) if, since last assessment, they avoided activities due to their pain if there was a perceived risk that it will hurt or make the current pain worse.

*Intraindividual Pain Variation Metrics.* All pain metrics were based on previously published research (Mun et al., 2019; Stone et al., 2021a, 2021b) and derived from pain intensity reports from the repeated EMA; pain metrics were calculated for those providing more than 1 response to allow for calculation of all metrics. At every repeated assessment, participants were asked to rate the intensity of their current pain, on a scale of 0 (*no pain*) to 10 (*pain as bad as it could be*). Dynamic pain metrics calculated from pain intensity reports included overall maximum pain level (0–10), overall minimum pain level (0–10), average pain level (0–10), % of time in high pain (≥ 7/10), % of time in low pain (≤ 3/10). Pain instability metrics included intraindividual standard deviation (iSD; individual coefficient of variability over the data collection period, meant to capture fluctuations in pain; Hyun et al., 2022), mean square of successive differences (MSSD; to integrate both magnitude of change and temporal dependency of observations; Jahng et al., 2008), and probability of acute change (PAC; clinical pain variability indicating the probability of either a decrease or increase in pain intensity scores of 2 or more from adjacent assessments; Berner et al., 2017).

### Procedure

The current manuscript represents a secondary data analysis from a previously published study. For detailed procedures, please see Rogers et al. (2024). Following a brief eligibility survey, participants completed a baseline assessment comprised of a brief interview and a battery of self-report questionnaires. Then, participants completed 5 daily surveys on their smartphone using the Smartphone Ecological Momentary Assessment (SEMA) mobile application (O’Brien et al., 2024). Surveys were administered between 9AM and 9PM for a total of 7 days (for up to 35 surveys per participant), and surveys expired 30 min after being sent if not completed. Compensation was based on survey completion rates, with 80% completion being associated with the highest rates of compensation.

### Data analytic plan

Analyses were conducted using *R Version 2024.04.2*. First, descriptive statistics and correlations among variables were examined. Then, pain variability metrics were calculated, in line with Mun et al. (2019), and descriptive statistics and correlations among variables were examined. Analyses were conducted in 2 stages. First, given the 3-level multi-level structure (assessments within days within people) of the longitudinal data, an “empty” random intercept model for each criterion variable (pain interference, pain-related negative affective, pain-related activity avoidance) was estimated to calculate the intra-class correlation (ICC) at each level of data; levels that accounted for at least 5% of variance in the outcome were retained in final analyses. Then, in line with recommendations for analyzing intensive longitudinal data as well as past work (Bolger, 2013; Rogers et al., 2022, 2024), to examine the impact of pain variability characteristics on longitudinal pain outcomes, a series of random intercept multi-level models (2-level and 3-level), controlling for study day, were run, with each variability metric included in a separate model to examine the contribution of each metric. To examine heterogeneity in the criterion variables, random intercepts were specified. Statistical significance level was set to *p* < 0.05.

## Results

### Descriptive statistics

**Fig. 1:**
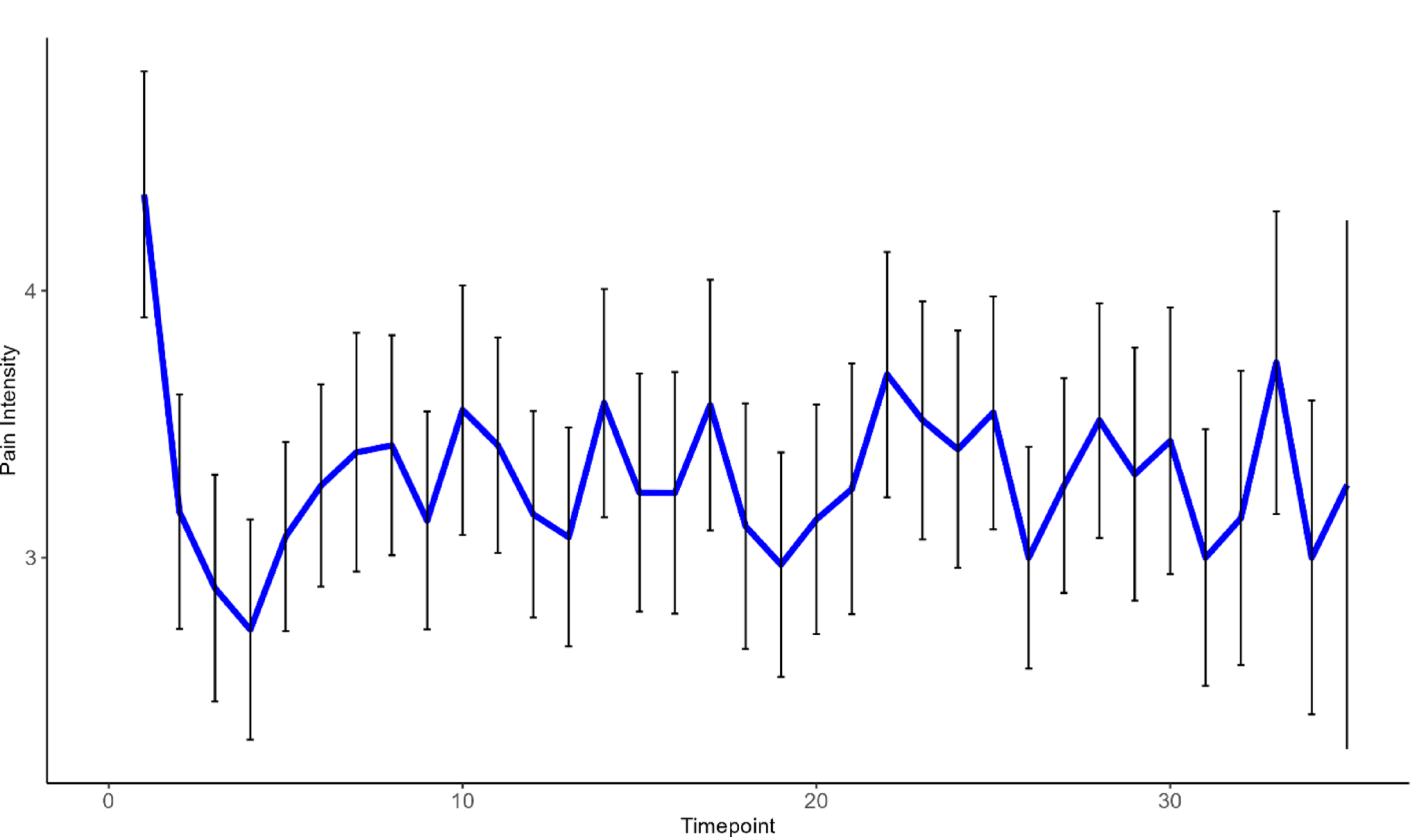
Variability in pain intensity over time

The current study included 48 (*M*_*age*_ = 24.75, *SD* = 5.69, 79% female) individuals with chronic pain who completed > 1 EMA prompt, with the mean number of EMAs completed = 24.75. For full descriptive statistics, see Table 1, and for means and correlations among variables, see Table 2. Further, patterns of pain intensity differed between participants (see Fig. 1 for example and ICC below for statistical differences).

**Table 1:** Participant characteristics (n = 48)

	N (%)
*Sex*	
Male	10 (21%)
Female	38 (79%)
*Race*	
White	21 (44%)
Black/African American	2 (4.2%)
Asian	16 (33%)
Native American/Alaskan Native	3 (6.3%)
Other	6 (13%)
*Ethnicity*	
Non-Hispanic/Latinx	32 (67%)
Hispanic/Latinx	16 (33%)
*Marital Status*	
Single	36 (75%)
Living with partner	6 (13%)
Married	4 (8.3%)
Divorced	1 (2.1%)
Separated	1 (2.1%)
*Income*	
< $25,000	39 (81.2%)
$25,000–$49,999	6 (12.5%)
$50,000–$74,999	2 (4.2%)
> $75,000	1 (2.1%)

**Table 2:** Variable means and correlations

	Mean	2	3	4	5	6	7	8	9	10	11
1. Pain-related Disability	2.61	0.76*	0.26*	0.60*	0.43*	0.43*	0.48*	− 0.55*	0.12*	0.13*	0.15*
2. Pain-related NA	2.90	–	0.23*	0.55*	0.45*	0.35*	0.48*	− 0.47*	0.17*	0.16*	0.16*
3. Pain-related Activity Avoidance	4.35		–	0.16*	0.22*	− 0.05	0.13*	− 0.04	0.26*	0.27*	0.21*
4. Mean Pain Intensity	3.41			–	0.64*	0.72*	0.73*	− 0.86*	0.13*	0.11*	0.23*
5. Highest Pain Level	6.77				–	0.18*	0.56*	− 0.46*	0.71*	0.64*	0.60*
6. Lowest Pain Level	1.17					–	0.44*	− 0.67*	− 0.43*	− 0.32*	− 0.21*
7. Time in High Pain	0.10						–	− 0.49*	0.22*	0.18*	0.16*
8. Time in Low Pain	0.41							–	− 0.06*	− 0.001	− 0.11*
9. Intraindividual Standard Deviation	1.63								–	0.87*	0.72*
10. MSSD	4.16									–	0.80*
11. PAC	0.18										–

*Indicates *p* < 0.05

### Intraindividual pain variability and functional behavior

*Pain-related Disability*. The empty model found that the ICC explained 46.4% of the variability in pain-related disability at the person level, and 4.2% of the variance was at the day level; these data suggest limited nesting at the day level, and that a 3-level model is not required (omitting the day level). Fixed effect estimates from separate models suggested that mean pain intensity (*b* = 0.89, *se* = 0.08, *p* < 0.001), highest pain level (*b* = 0.22, *se* = 0.04, *p* < 0.001), lowest pain level (*b* = − 0.14, *se* = 0.02, *p* < 0.001), time in high pain (*b* = 7.87, *se* = 1.37, *p* < 0.001), and time in low pain (*b* = -4.92, *se* = 0.63, *p* < 0.001) were statistically significantly associated with pain-related disability (see Table 3 bold indicates statistically significant values (*p* < 0.05)). There were no statistically significant associations for iSD, PAC, or MSSD.

**Table 3:** Fixed effect regression parameter estimates

	Pain-related disability			Pain-related negative affect			Pain activity avoidance		
	*B*	*SE*	*p-value*	*B*	*SE*	*p-value*	*B*	*SE*	*p-value*
*Mean Pain Intensity*									
Mean Pain Intensity	**0.89**	**0.08**	< 0.001*	**0.89**	**0.10**	< 0.001*	0.13	0.49	0.30
Study Day	**0.02**	**0.01**	0.01*	**0.11**	**0.03**	< 0.001*	− 0.03	0.02	0.13
*Highest Pain Level*									
High Pain	**0.46**	**0.09**	< 0.001*	**0.53**	**0.10**	< 0.001*	0.16	0.68	0.08
Study Day	**0.02**	**0.01**	0.01*	**0.11**	**0.03**	< 0.001*	− 0.03	0.02	0.14
*Lowest Pain Level*									
Low Pain	**0.73**	**0.13**	< 0.001*	**0.71**	**0.16**	< 0.001*	− 0.10	0.14	0.46
Study Day	**0.02**	**0.01**	0.01*	**0.11**	**0.03**	< 0.001*	− 0.03	0.02	0.13
*% of Time in High Pain*									
Time in High Pain	**7.84**	**1.36**	< 0.001*	**8.35**	**1.52**	< 0.001*	1.87	1.48	0.21
Study Day	**0.02**	**0.01**	0.01*	**0.11**	**0.03**	< 0.001*	− 0.03	0.02	0.13
*% of Time in Low Pain*									
Time in Low Pain	− **4.87**	**0.63**	< 0.001*	− **4.50**	**0/83**	< 0.001*	− 0.04	0.81	0.96
Study Day	**0.02**	**0.01**	0.01*	**0.11**	**0.03**	< 0.001*	− 0.03	0.02	0.13
*Intraindividual Standard Deviation*									
iSD	0.30	0.37	0.42	0.05	0.40	0.20	**0.62**	**0.29**	0.038*
Study Day	**0.02**	**0.01**	0.01*	**0.11**	**0.03**	< 0.001*	− 0.03	0.02	0.13
*Mean Square of Successive Differences*									
MSSD	0.07	0.06	0.26	0.10	0.07	0.15	**0.11**	**0.05**	0.02*
Study Day	**0.02**	**0.01**	0.01*	**0.11**	**0.03**	< 0.001*	− 0.03	0.02	0.13
*Probability of Acute Change*									
PAC	1.57	1.20	0.20	1.96	1.28	0.14	1.71	0.96	0.08
Study Day	**0.02**	**0.01**	0.01*	**0.11**	**0.03**	< 0.001*	− 0.03	0.02	0.13

*Pain-related negative affect* The empty model indicated that the ICC explained 49.4% of the variability in pain-related negative affect at the person level, and 4.8% of the variance was at the day level; again suggesting that a 3-level model is not required (omitting the day level). Fixed effect estimates from separate models suggested that mean pain intensity (*b* = 0.89, *se* = 0.10, *p* < 0.001), highest pain level (*b* = 0.24, *se* = 0.04, *p* < 0.001), lowest pain level (*b* = − 0.12, *se* = 0.02, *p* < 0.001), time in high pain (*b* = 8.35, *se* = 1.52, *p* < 0.001), and time in low pain (*b* = − 4.50, *se* = 0.82, *p* < 0.001) were statistically significantly associated with pain-related negative affect (see Table 3). There were no statistically significant associations for iSD, PAC, or MSSD.

*Pain-related activity avoidance* The empty model found that the ICC accounted for 67.3% of the variability in pain-related activity avoidance was at the person level, and 6.6% of the variance was at the day level; evidence in favor of a 3-level model. Fixed effect parameter estimates suggested that iSD (*b* = 0.62, *se* = 0.29, *p* = 0.038), MSSD (*b* = 0.13, *se* = 0.05, *p* = 0.017), and PAC (*b* = 3.90, *se* = 1.78, *p* = 0.034) were associated with pain-related activity avoidance (see Table 3). No statistically significant effects were evident for mean, highest, or lowest pain level, time in high pain, or time in low pain on pain-related activity avoidance.

### Sensitivity analyses

All models were re-run including mean pain intensity levels. Despite high correlations between mean pain intensity levels and outcomes (> 0.7), the patterns of findings where dynamic metrics were associated with pain-related disability and negative affect, and instability metrics was associated with activity avoidance, did not change when including mean pain intensity, suggestive of the relatively importance of variability metrics on outcomes.

## Discussion

Considering pain as a dynamic process is an important, yet relatively under-explored, aspect of pain that can provide insight into nature of pain and its comorbidities (de Koning et al., 2018; Han et al., 2024; Mun et al., 2019; Tupper et al., 2013). The current study examined the impact of intraindividual pain variation metrics on pain-related disability, pain-related negative affect, and pain-related activity avoidance.

Data for the primary analysis was largely in line with expectations. Specifically, mean pain, highest pain, lowest pain, and percent of time in high and low pain were associated with pain-related disability and pain-related negative affect (dynamic metrics), whereas intraindividual standard deviation, mean square of successive differences were associated with activity avoidance (instability metrics); these findings suggest that there may be an important conceptual difference between pain *instability* and variability generally. The Fear-Avoidance model of chronic pain (Vlaeyen & Linton, 2012) suggests that activity avoidance is associated with greater chronic pain symptoms and pain-related disability. The current data contribute to this model by suggesting variability in pain experience, or what clinically could be referred to as pain unpredictability (Pavy et al., 2024a), is related to constructs often associated with pain avoidance (i.e., pain-related disability and negative affect; Meulders, 2019; Wideman et al., 2013; Zale & Ditre, 2015). Future research could build on our findings by exploring pain variability in terms of pain-related avoidance. Instability, as measured by multiple metrics, was consistent in its association with pain-related activity avoidance, highlighting that the unpredictable nature of pain may be the most clinically salient when examining activity avoidance (Yoshida et al., 2013).

The results of this study should be considered preliminary in need of replication but, when coupled with recent theoretical and empirical work (Mun et al., 2019; Schneider et al., 2021; Stone et al., 2021a, 2021b), fill an important gap in the literature. Relying on a retrospective, average pain score alone does not provide an adequate characterization of pain and the way it may impact those with chronic pain (Ballantyne & Sullivan, 2015). The present findings are consistent with previous work (Gillving et al., 2022; Harris et al., 2005; Tupper et al., 2013) in suggesting that intraindividual pain variability is important to numerous aspects of pain experience. Measuring and addressing pain variability may be important to treatment success, by specifically targeting pain predictability and expectancies that have clinically meaningful associations with functional outcomes (Pavy et al., 2024b). Here, it may be useful for adults with chronic pain to complete a daily diary of their pain symptoms prior to initiating treatment to understand their intraindividual pain variation and its impact on pain and functioning (work, social), and leverage this to provide treatments more tailored to the individual. For example, persons with higher levels of pain *instability* (and thus at risk for pain-related activity avoidance) may benefit directly from exposure (Macedo et al., 2010), whereas those who spend a large percentage of time in high pain may benefit from a more cognitive approach (Cheng & Cheng, 2019; Otis, 2007).

The current study is not without limitations. First, while everyone in the sample identified experiencing chronic pain, the sample was not treatment-seeking, did not undergo a comprehensive pain diagnostic evaluation, and are limited in variability in demographic and cultural factors, which may not be reflective of all persons with chronic pain. Similarly, demographic assessment of functional impairment (e.g., work performance) was not specifically anchored to pain, and therefore it was unclear the degree to which pain impacted work/school functioning. Therefore, replication of the current findings on different samples of individuals with chronic pain, with more specific assessment of functional impairment across multiple domains is warranted. Second, the measure of pain-related negative affect assessed multiple emotions together, in one item, limiting understanding of how specific emotions may be related to variability metrics. It may be possible that, if parsed out into individual emotions in a future study, the impact of variability may have been different and is worth exploring in future research given the documented associated with negative emotions and pain (Lumley et al., 2011).

Third, it is important to discuss potential limitations of variability calculations. Previous research indicates that estimates of pain variability are influenced, in part, by mean pain level (Mun et al., 2019; Schneider et al., 2021), but in the current study, the correlations between mean pain level and variability metrics would have likely resulted in issues of multi-collinearity. Future research, replicating the current findings and utilizing alternative statistical approaches to account for both mean level and variability metrics (e.g., mixed effects location scale models; Dzubur et al., 2020) would be an important next step. Additionally, while the current study utilized established cut-points for “high” and “low” pain, there are inherent limitations to this approach that does not account for individual differences in pain experience (e.g., ceiling effects, reporting bias) that may influence results. Furthermore, while not testable in the current study, it is possible that a sequential model exists that incorporates aspects of pain variability as well as pain-related activity avoidance, disability, and negative affect, which requires significant within-day variability and a study designed to capture time-lagged relationships The pain processing model (Riley et al., 2000) is one empirical model that could be tested in future work to disentangle the sequential order of these relationships. Finally, the current study assessed individuals over 7 days and focused on variability metrics in pain intensity. While there is a large amount of data that was captured, it is possible that patterns of variability (and their associations with pain outcomes, both within- and between-person and day) may differ when assessing pain over a longer period, or when assessing variability in functional impairment over time. Future research should examine the *optimal* number and type (e.g., intensity vs. interference) of assessments of pain needed to both balance patient burden as well as providing the necessary information for clinical care.

Overall, the current study provides empirical support for the importance of pain variability in conceptualizing pain experience and factors that lead to pain-related functional impairment. Moving towards understanding pain as a dynamic experience has the potential to improve our treatments and reduce the negative impact of chronic pain.
